# Once weekly selinexor, carfilzomib and dexamethasone in carfilzomib non-refractory multiple myeloma patients

**DOI:** 10.1038/s41416-021-01608-2

**Published:** 2021-11-20

**Authors:** Cristina Gasparetto, Gary J. Schiller, Sascha A. Tuchman, Natalie S. Callander, Muhamed Baljevic, Suzanne Lentzsch, Adriana C. Rossi, Rami Kotb, Darrell White, Nizar J. Bahlis, Christine I. Chen, Heather J. Sutherland, Sumit Madan, Richard LeBlanc, Michael Sebag, Christopher P. Venner, William I. Bensinger, Noa Biran, Sonia Ammu, Osnat Ben-Shahar, Andrew DeCastro, Dane Van Domelen, Tianjun Zhou, Chris Zhang, Ohad S. Bentur, Jatin Shah, Sharon Shacham, Michael Kauffman, Brea Lipe

**Affiliations:** 1 Duke University Medical Center, Durham, NC USA; 2 David Geffen School of Medicine at UCLA, Los Angeles, CA USA; 3 University of North Carolina, Chapel Hill, NC USA; 4 Carbone Cancer Center, University of Wisconsin-Madison, Madison, WI USA; 5 University of Nebraska Medical Center, Omaha, NE USA; 6 Colombia University, New York, NY USA; 7 NYPH Weill Cornell, New York, NY USA; 8 Cancer Care Manitoba, Winnipeg, MB Canada; 9 Dalhousie University and Queen Elizabeth II Health Sciences Centre, Halifax, NS Canada; 10 Charbonneau Cancer Research Institute, Calgary, AB Canada; 11 Department of Medical Oncology and Hematology, Princess Margaret Cancer Centre, University of Toronto, Toronto, ON Canada; 12 Vancouver General Hospital, Vancouver, BC Canada; 13 Banner MD Anderson Cancer Center, Gilbert, AZ USA; 14 Maisonneuve-Rosemont Hospital, University of Montreal, Montreal, QC Canada; 15 Royal Victoria Hospital, Montreal, QC Canada; 16 Cross Cancer Institute, University of Alberta, Edmonton, AB Canada; 17 Myeloma and Transplant Program, Swedish Cancer Institute, Seattle, WA USA; 18 Hackensack Meridian Health, Hackensack University Medical Center, Teaneck, USA; 19 Karyopharm Therapeutics Inc., Newton, MA USA; 20 University of Rochester Medical College, Rochester, NY USA

**Keywords:** Myeloma, Drug development

## Abstract

**Background:**

Proteasome inhibitors (PIs), including carfilzomib, potentiate the activity of selinexor, a novel, first-in-class, oral selective inhibitor of nuclear export (SINE) compound, in preclinical models of multiple myeloma (MM).

**Methods:**

The safety, efficacy, maximum-tolerated dose (MTD) and recommended phase 2 dose (RP2D) of selinexor (80 or 100 mg) + carfilzomib (56 or 70 mg/m^2^) + dexamethasone (40 mg) (XKd) once weekly (QW) was evaluated in patients with relapsed refractory MM (RRMM) not refractory to carfilzomib.

**Results:**

Thirty-two patients, median prior therapies 4 (range, 1–8), were enrolled. MM was triple-class refractory in 38% of patients and 53% of patients had high-risk cytogenetics del(17p), t(4;14), t(14;16) and/or gain 1q. Common treatment-related adverse events (all/Grade 3) were thrombocytopenia 72%/47% (G3 and G4), nausea 72%/6%, anaemia 53%/19% and fatigue 53%/9%, all expected and manageable with supportive care and dose modifications. MTD and RP2D were identified as selinexor 80 mg, carfilzomib 56 mg/m^2^, and dexamethasone 40 mg, all QW. The overall response rate was 78% including 14 (44%) ≥ very good partial responses. Median progression-free survival was 15 months.

**Conclusions:**

Weekly XKd is highly effective and well-tolerated. These data support further investigation of XKd in patients with MM.

## Introduction

Exportin 1 (XPO1) is a critical nuclear exporter for tumour suppressor proteins (TSPs, e.g. p53, IκB and FOXO3a) [1–3] and eIF4E-bound oncoprotein messenger RNAs (mRNAs) (e.g. c-Myc, Bcl-xL, MDM2 and cyclin D1) [1, 2, 4, 5]. XPO1 is overexpressed in many cancers including multiple myeloma (MM), and this overexpression of XPO1 enables cancer cells to escape TSP-mediated cell cycle arrest and apoptosis [1, 2, 6, 7]. Indeed, XPO1 overexpression correlates with poor prognosis of cancer patients and drug resistance of their disease [1, 2, 6, 8, 9].

Selinexor is a novel, first-in-class oral selective inhibitor of nuclear export (SINE) compound that blocks XPO1, forcing the nuclear retention and activation of TSPs, ultimately causing cancer cell death [1]. Preclinical data demonstrate that selinexor reactivates multiple TSPs relevant to MM, inhibits nuclear factor-κB activity, reduces c-Myc levels and reactivates glucocorticoid receptor signalling in the presence of dexamethasone, all of which has the downstream effect of suppressing MM cell growth [1–3, 10]. Consistent with these preclinical results, in the STORM (Selinexor Treatment of Refractory Myeloma) clinical trial in patients with MM refractory to at least one proteasome inhibitor (PI), one immunomodulatory agent (IMiD) and daratumumab (triple-class refractory), selinexor 80 mg and dexamethasone 20 mg, both twice weekly, induced a 26.4% overall response rate (ORR) and 4.4-month median duration of response (DOR) [11]. Based on these results, selinexor received accelerated approval from the Food and Drug Administration (FDA) for patients with relapsed refractory MM (RRMM) [12, 13].

The combination of nuclear export and proteasome inhibition is synergistic in preclinical models, wherein PIs prevent the proteasomal degradation of TSPs and SINE compounds force their nuclear retention, leading to apoptosis of cancer cells [1, 14]. Importantly, elevated levels of XPO1 can mediate PI resistance by exporting TSPs from the nucleus [15, 16]. Thus, XPO1 inhibition can overcome PI resistance in MM cells in cell lines as well as in patient-derived MM cells ex vivo [8, 14, 17], and selinexor was shown to sensitise PI-refractory MM cells to bortezomib and carfilzomib [16]. These preclinical results were confirmed in phase 3 BOSTON (Bortezomib, Selinexor, and Dexamethasone in Patients With Multiple Myeloma) trial, where *once* weekly (QW) selinexor, bortezomib and dexamethasone (XVd) showed superior median progression-free survival (PFS; 13.9 vs. 9.5 months) and ORR (76.4 vs. 62.3%), reduced peripheral neuropathy and a trend to reduced mortality, as compared with standard *twice* weekly bortezomib and dexamethasone (Vd), despite XVd using 40% less bortezomib and 25% less dexamethasone than standard Vd [18]. The BOSTON trial led to an FDA approval of XVd for adult patients with MM who have received at least one prior therapy [12].

Carfilzomib, a second-generation PI that is more potent than bortezomib [19–21], is approved for the treatment of MM with dexamethasone and in combination with other agents [22]. Selinexor and carfilzomib showed marked synergistic anti-tumour activity in various preclinical models in vitro and in vivo [17, 23]. A phase I trial in patients with RRMM validated these preclinical results, demonstrating that the combination of selinexor and carfilzomib with low-dose dexamethasone (XKd) is tolerable and is effective in inducing responses in heavily pretreated MM, including in disease refractory to carfilzomib [24]. The present trial was conducted to further validate these results and to show that weekly XKd is safe and tolerable and derives durable responses in patients whose MM is not refractory to carfilzomib.

## Methods

### Study design and oversight

This trial is part of the multi-arm Phase 1b/2 STOMP (Selinexor and Backbone Treatments of Multiple Myeloma Patients) study evaluating the safety and efficacy of selinexor in combination with FDA-approved therapies for RRMM (ClinicalTrials.gov #NCT02343042). Here, we report data from the dose-evaluation and dose-expansion phases of varying doses of XKd. The primary objectives of the dose-evaluation phase were to determine the maximum-tolerated dose (MTD), safety and tolerability, and to identify the recommended phase 2 dose (RP2D) for the XKd regimen.

Patients with previously treated MM that was not *refractory* specifically to carfilzomib (but may have had prior PI treatment), and who had not received prior selinexor, were eligible for enrollment. Refractory disease and high-risk disease were defined per International Myeloma Working Group (IMWG) guidelines as lack of at least a minimal response (MR) while on therapy, or disease progression within 2 months of completing therapy [25, 26]. A full list of inclusion/exclusion criteria has been published previously [27].

The study protocol was approved by the institutional review board or an independent ethics committee at each participating centre and was in accordance with the Declaration of Helsinki, the International Conference on Harmonisation-Good Clinical Practice and local laws. All patients provided written informed consent prior to enrollment. All authors reviewed the data for accuracy and collaborated in the preparation of the manuscript.

### Treatments

In the dose-evaluation phase, the starting selinexor dose was 100 mg QW, carfilzomib 20/56 mg/m^2^ QW (20 mg/m^2^ only on C1D1 and 56 mg/m^2^ thereafter, dosed on days 1, 8 and 15, of 28-day cycles) and 40 mg QW dexamethasone (Table 1). Rules for dose level evaluation vs. reduction followed standard 3 + 3 rules. As selinexor and carfilzomib antineoplastic effects synergise, and as selinexor is typically used at 60, 80 or 100 mg QW, and carfilzomib 45–70 mg/m^2^ weekly, optimising dosing of the combination requires modulating both agents. To be conservative, we chose either the MTD of selinexor (i.e. 100 mg—Bahlis et al. [27]) with one level down from MTD of carfilzomib QW (i.e. 56 mg/m^2^; Berenson et al. [28]) or one level down from MTD of selinexor (i.e. 80 mg) with the MTD of carfilzomib QW (i.e. 70 mg/m^2^); the step-down dose involved one level down from MTDs of both selinexor (i.e. 80 mg) and carfilzomib (i.e. 56 mg/m^2^). Ultimately, we will choose the dose with the highest benefit/risk ratio.

**Table 1:** Treatment schedule, dose levels and DLTs.

Dose levels	Selinexor, days 1, 8, 15 and 22	Dexamethasone, days 1, 8, 15 and 22	Carfilzomib, days 1, 8, and 15	Patients enrolled	Patients DLT evaluable	Patients with DLT	DLTs
1	100 mg PO	40 mg IV or PO	56 mg/m^2^ IV	3	2^a^	2^b^	Selinexor dose reduction due to Grade 3 thrombocytopenia; selinexor dose reduction due to Grade 3 vomiting
−1	80 mg PO	40 mg IV or PO	56 mg/m^2^ IV	6	6^c^	0	No DLTs
−1a	80 mg PO	40 mg IV or PO	70 mg/m^2^ IV	3	3	2^c^	Grade 4 thrombocytopenia and Grade 3 pneumonia; Grade 4 thrombocytopenia
−1b	60 mg PO	40 mg IV or PO	70 mg/m^2^ IV	3	ND	ND	ND
n1	100 mg PO—not on Day 22	40 mg IV or PO	56 mg/m^2^ IV	2	ND	ND	ND
n−1	80 mg PO—not on Day 22	40 mg IV or PO	70 mg/m^2^ IV	3	ND	ND	ND

*PO* oral, *IV* intravenous, *DLT* dose-limiting toxicities, *ND* not determined, *QW* once weekly.

^a^One patient was not DLT evaluable because the platelet count was <50 × 10^9^/L on C1D1.

^d^One patient had dose reduction of selinexor (due to Grade 3 thrombocytopenia without bleeding) to 80 mg in cycle 1 and then to 60 mg (cycle 2) continuing with treatment through C13; the other had dose reduction of selinexor (due to Grade 3 vomiting) to 80 mg in cycle 1 and of carfilzomib to 37.3 mg/m2 continuing with treatment through C5.

^c^Patients enrolled after the first six patients were not included in the DLT assessment.

^b^One patient had dose reduction of selinexor to 60 and then to 40 mg, and of carfilzomib to 56 mg/m^2^ continuing with treatment through cycle 16. The other had dose reduction of selinexor to 60, 40 and finally 20 mg and of carfilzomib to 56 mg/m^2^ by further reductions continuing with treatment through week 3 of C26.

Dose-limiting toxicities (DLTs) were evaluated only in patients enrolled during the dose-evaluation phase over their first cycle of treatment. DLTs were defined as any of the following: (1) missing ≥25% of scheduled doses, a dose reduction or discontinuation due to treatment-related adverse events (TRAEs); (2) occurrence of Grade ≥3 nausea, vomiting, dehydration, diarrhoea or fatigue lasting >3 days despite optimal supportive care medications, or Grade 4 of these adverse events (AEs); (3) any other Grade 3 or 4 non-haematologic toxicity; (4) febrile neutropenia, Grade 4 neutropenia or Grade 4 thrombocytopenia lasting >7 days, and Grade ≥3 thrombocytopenia with clinically significant bleeding, petechiae or purpura.

Patients received 5-hydroxytryptamine-3 (5-HT3) antagonist (ondansetron 8 mg or equivalent, or an alternative if 5-HT3 antagonists were not tolerated) before each dose of selinexor and continued 2–3 times daily for at least 2 days. Additional supportive care was included as part of the protocol (see supplementary materials for full AE management guidelines).

### Study assessments and statistics

Efficacy was assessed using modified IMWG guidelines [29]. ORR was defined as the percentage of patients who achieved a confirmed partial response (PR) or better before the progressive disease (PD) or initiating a new anti-MM treatment; clinical benefit rate was defined similarly but included MR. PFS was defined as the duration from the first dose of study treatment to the first confirmed PD or death due to any cause. DOR was defined for responders only as the duration from first PR or better to first confirmed PD or death due to any cause. For PFS and DOR, patients who discontinued treatment prior to confirmed PD or death, or who were still on treatment with no confirmed PD at the time of the data extract, were censored at the latest response assessment on or before the date of treatment discontinuation (where applicable). Overall survival (OS) was defined as the duration from the first dose of study treatment to death due to any cause. Survival endpoints were analysed using Kaplan–Meier methodology; median follow-up times were estimated using the reverse Kaplan–Meier method [30].

Safety was monitored throughout the study and severity was assessed according to the National Cancer Institute Common Terminology Criteria for Adverse Events, v4.03. All patients who received at least a single dose of study medication were included in safety and efficacy analyses.

The sample size for the dose-evaluation phase of the study was based on the standard 3 + 3 dose-escalation scheme. The expansion phase was designed to test the null hypothesis that the true ORR was ≤30% against a one-sided alternative and required a sample size of 20 patients, where the first 10 patients treated at the RP2D were considered the first stage of the two-stage design. If ≤3 patients responded in Stage 1, the expansion phase would be terminated. If ≥4 patients responded, an additional ten patients were to be enrolled to include a total of 20 patients at the RP2D. If the total number of patients responding was ≥9, the treatment would be accepted as promising for further study. Assuming a true ORR of 55%, this design achieved at least 80% power at a one-sided 0.10 significance level.

## Results

### Patients and treatment

A total of 32 patients were enrolled between March 2018 and Feb 2021: 3 patients in the selinexor 100 mg with 56 mg/m^2^ carfilzomib cohort, 3 in the selinexor 80 mg with 70 mg/m^2^ carfilzomib cohort, 18 in the selinexor 80 mg with 56 mg/m^2^ carfilzomib cohort, 3 in the selinexor 60 mg with 70 mg/m^2^ carfilzomib cohort, and 2 and 3 in the selinexor 60 mg with 56 or 70 mg/m^2^ carfilzomib, respectively, cohorts in which selinexor was given on days 1, 8 and 15, but not on day 22 of the 4-week cycle (Table 1). The ‘stepped down’ selinexor 60 mg QW dose combined with 70 mg/m^2^ carfilzomib cohort and the cohorts in which selinexor was administered only on weeks 1–3 of the 4-week cycle were exploratory dose-evaluation cohorts initiated to test selinexor with the currently approved once-weekly carfilzomib dose (i.e. 70 mg/m^2^ QW) or to test the impact of 3/4-week dosing of selinexor on safety and tolerability.

The median age was 69.5 years (range 35–76 years), 63% were males and the median number of prior therapies was 4 (range 1–8). All patients had been treated with bortezomib and at least one IMiD, 3 (9%) had previously received carfilzomib, 59% had MM refractory to a PI, 75% had MM refractory to an IMiD and 66% had MM refractory to anti-CD38 monoclonal antibody (mAb). In addition, 50% of the patients had MM refractory to both a PI and an IMiD, and 38% had MM refractory to a PI, an IMiD and an anti-CD38 mAb. Fifty-three per cent of the patients had high-risk cytogenetics, defined as del 17p, t(4;14), t(14;16) or gain of 1q (3 or 4 fold). Patient demographics and disease characteristics at baseline are shown in Table 2.

**Table 2:** Demographics and clinical characteristics.

Characteristics	All patients (*n* = 32)
Median age, years (range)	69.5 (35–76)
Age, years, *N* (%)	
≤50	2 (6.3)
51–64	8 (25.0)
65–74	19 (59.4)
≥75	3 (9.4)
Male, *N* (%)	20 (62.5)
ECOG performance status, *N* (%)	
0	8 (25.0)
1	22 (68.8)
2	2 (6.3)
Median no. of years since diagnosis (range)^a^	5.25 (0.4–11.3)
ISS stage at initial diagnosis, *N* (%)	
I	5 (15.6)
II	10 (31.3)
III	4 (12.5)
Unknown	13 (40.6)
Median no. of prior therapies (range)	4 (1–8)
Prior therapies, treated: refractory^b^, *N* (%)	
Bortezomib	32 (100.0):14 (43.8)
Carfilzomib	3 (9.4):1 (3.1)
Ixazomib	11 (34.4):7 (21.9)
Oprozomib	2 (6.3):1 (3.1)
Thalidomide	3 (9.4):0 (0)
Lenalidomide	31 (96.9):18 (56.3)
Pomalidomide	23 (71.9):18(56.3)
Daratumumab	22 (68.8):21 (65.6)
Belantamab mafodotin	1 (3.1):1 (3.1)
Elotuzumab	10 (31.3):6 (18.8)
PIs^c^	32 (100.0):19 (59.4)
IMiDs^d^	32 (100.0):24 (75.0)
Anti-CD38 mAb^e^	22 (68.8):21 (65.6)
PI and IMiD and Anti-CD38 mAb^f^	22 (68.8):12 (37.5)
≥2 PIs and ≥2 IMiDs and anti-CD38 mAb	9 (28.1):3 (9.4)
Autologous stem-cell transplantation, *N* (%)	23 (71.9)
Genetic abnormalities at initial diagnosis or screening, *n* (%)	
del(17p)	9 (28.1)
t(4;14)	7 (21.9)
t(14;16)	4 (12.5)
Gain 1q	4 (12.5)
Any of del(17p), t(4;14), t(14;16) or gain 1q	17 (53.1)

^a^Years from the initial diagnosis to C1D1 start date. Fourteen patients had incomplete diagnosis dates, which were imputed as the first day of the year if the month and day were missing, and as the first day of the month if only the day was missing.

^b^Refractory is defined as prior treatment meeting refractory criteria (best overall response of PD or SD, or disease progression during treatment or within 60 days of the end of treatment, or end of treatment within 60 days of the first dose in STOMP), and no subsequent treatment with PR or better and no refractory criteria met.

^c^PIs (proteasome inhibitors), include bortezomib, carfilzomib, ixazomib: number of patients who were treated with at least one and were refractory to at least one.

^d^IMiDs (immunomodulatory imide drugs) include thalidomide, lenalidomide and pomalidomide. A number of patients were treated with at least one and were refractory to at least one.

^e^Anti-CD38 mAb include daratumumab and isatuximab. A number of patients were treated with at least one of the two drugs and were refractory to at least one.

^f^PI and IMiD and Anti-CD38 mAb, patients who were treated with at least one PI, one immunomodulatory drug and one anti-CD38 mAb and were refractory to at least one of each.

As of the cut-off date of 11 May 2021, 10 (31.3%) patients were still receiving treatment, 12 (38%) patients discontinued due to PD, 5 (15.6%) discontinued due to AEs (cardiac failure; peripheral neuropathy; left ventricular failure; upper respiratory tract infection + elevated creatinine; atrial fibrillation + dyspnoea + dizziness), 1 patient discontinued due to drug toxicity, 1 patient withdrew consent, 1 patient died (respiratory failure not related to study drugs) and 1 patient discontinued to undergo autologous haematopoietic stem-cell transplantation.

### Efficacy

**Fig. 1:**
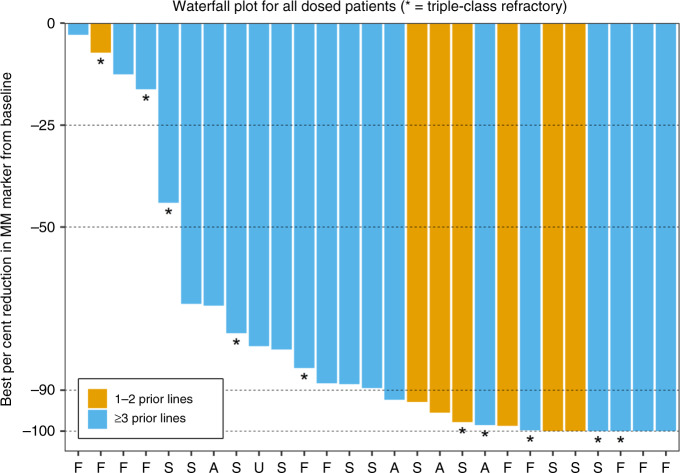
Depth of response to XKd in patients with relapsed or refractory multiple myeloma. Waterfall plot depicts the best % changes in the IgA, primary myeloma marker (serum M-protein, urine M-protein or difference between involved and uninvolved serum-free light-chain levels) from baseline. F free light chain, S SPEP, U UPEP, A IgA.

**Fig. 2:**
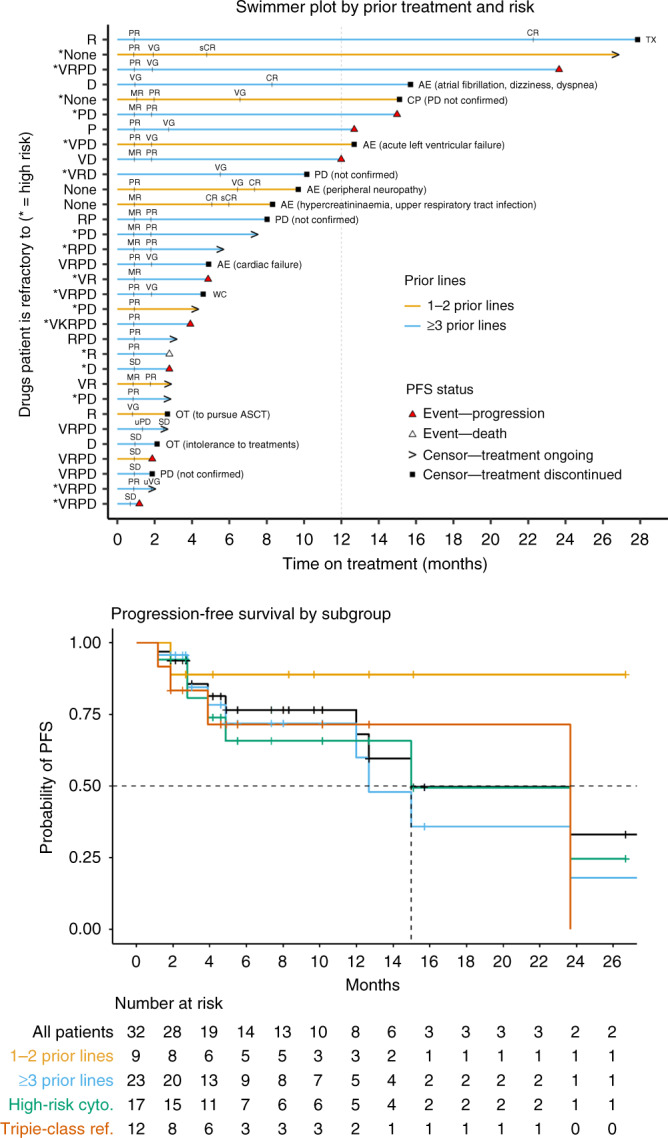
Progression Free Survival. **a** Time to PFS event or censor for all dosed patients. PFS event is confirmed disease progression or death before documented treatment discontinuation or the start of a new anti-MM treatment. *Y*-axis labels indicate which drugs each patient was refractory to (V bortezomib, K carfilzomib, R lenalidomide, P pomalidomide, D daratumumab) and whether they had high-risk cytogenetics (* = Yes), defined as any of del(17p), t(4;14), t(14;16) or gain 1q at initial diagnosis or screening. TX toxicity to study drug, AE adverse event, CP disease progression (clinical progression), WC withdrawal of consent, OT other, event PFS event. **b** Kaplan-Meier analysis of progression-free survival (PFS) for all dosed patients (*n* = 32). Median PFS for all patients was 15.0 months (95% CI, 12.0–NE) and 23.7 months (95% CI, 3.9–NE) patients with triple-class refractory MM.

The response was evaluated in all 32 patients. The overall ORR was 78.1% (25/32): 2 (6.3) stringent complete responses (sCRs), 3 (9.4%) CR, 9 (28.1%) very good PRs (VGPRs) and 11 (34.4%) PR. One (3.1%) additional patient achieved an MR (Table 3 and Fig. 1). PFS for individual patients is presented in Fig. 2a, and the Kaplan–Meier curve is shown in Fig. 2b. The overall median PFS was 15.0 months (95% confidence interval (CI), 12.0–NE; median follow-up 8.0 months), the median DOR was 22.7 months (95% CI, 11.8–NE; median follow-up 5.6 months) and the median OS (mOS) was not reached (95% CI, NE–NE; median follow-up 15.1 months). In the nine patients with ≤2 prior therapies, the ORR was 88.9%.

**Table 3:** Efficacy.

Group	*N*	*N* (%)							
		ORR^a^	CBR^b^	sCR	CR	VGPR^c^	PR	MR	SD
Overall	32	25 (78.1)	26 (81.3)	2 (6.3)	3 (9.4)	9 (28.1)	11 (34.4)	1 (3.1)	6 (18.8)
Prior lines of therapy									
1–2	9	8 (88.9)	8 (88.9)	2 (22.2)	1 (11.1)	3 (33.3)	2 (22.2)	0 (0.0)	1 (11.1)
≥3	23	17 (73.9)	18 (78.3)	0 (0.0)	2 (8.7)	6 (26.1)	9 (39.1)	1 (4.3)	5 (21.7)
Triple-class status									
Not triple-class exposed	10	10 (100.0)	10 (100.0)	2 (20.0)	2 (20.0)	3 (30.0)	3 (30.0)	0 (0.0)	0 (0.0)
Triple-class exposed	22	15 (68.2)	16 (72.7)	0 (0.0)	1 (4.5)	6 (27.3)	8 (36.4)	1 (4.5)	6 (27.3)
Triple-class refractory	12	8 (66.7)	8 (66.7)	0 (0.0)	0 (0.0)	6 (50.0)	2 (16.7)	0 (0.0)	4 (33.3)
High-risk cytogenetics^d^									
Yes	17	14 (82.4)	15 (88.2)	1 (5.9)	0 (0.0)	6 (35.3)	7 (41.2)	1 (5.9)	2 (11.8)
No	15	11 (73.3)	11 (73.3)	1 (6.7)	3 (20.0)	3 (20.0)	4 (26.7)	0 (0.0)	4 (26.7)

Note: Responses were investigator reported and internally assessed according to the International Myeloma Working Group criteria.

*CBR* clinical benefit rate, *MR* minimal response, *ORR* overall response rate, *PD* progressive disease, *PR* partial response, *SD* stable disease, *VGPR* very good partial response.

^a^Overall response rate is the proportion of patients who achieved a partial response or better, before disease progression or initiating a new MM treatment.

^b^Clinical benefit rate is the proportion of patients who achieved minimal response or better, before disease progression or initiating a new MM treatment.

^c^One very good partial response was unconfirmed.

^d^Defined as any of del(17p), t(4;14), t(14;16), or gain 1q at initial diagnosis or screening.

XKd induced responses in patients with triple-class refractory MM (*n* = 12): ORR was 66.7% (8/12), the median PFS was 23.7 months (95% CI, 3.9–NE), the median DOR was 22.7 months (95% CI, NE–NE) and the mOS was 20.4 months (95% CI, 20.4–NE). Likewise, the presence of high-risk genetics [i.e. del(17p), t(4;14), t(14;16) or gain 1q] at initial diagnosis or screening did not compromise the efficacy of XKd: In the 17 patients with high-risk cytogenetics, the ORR was 82.4% (14/17), the median PFS was 15.0 months (95% CI, 4.9–NE), the median DOR was 22.7 months (95% CI, 13.1–NE) and the mOS was not reached (95% CI, 20.4–NE).

XKd induced rapid responses, 16 of the 25 responders achieved PR or better within the first cycle of treatment and 23 had ≥PR within the second cycle.

## Discussion

The aim of the present research was to determine the MTD, RP2D, safety and efficacy of weekly XKd for the treatment of RRMM. Although numbers were relatively small in this phase 2 study, across all doses tested, weekly XKd demonstrated high rates of anti-MM activity against heavily pretreated RRMM (1–8 prior therapies, median = 4), with an ORR of 78.1% including 2 (6.3%) sCR, 3 (9.4%) CR, 9 VGPR (28.1%) and 11 PR (34.4%). Moreover, XKd induced long-lasting responses with median DOR of 22.7 months, median PFS of 15.0 months and median OS not reached (median follow-up 15.1 months). No clinically significant and unexpected cumulative toxicities have been observed. Importantly, efficacy was preserved in genetically high-risk disease; ORR was 82.4%, median PFS 15.0 months, median DOR 22.7 months and median OS not reached. Similar results were observed in triple-class refractory MM where ORR was 66.7%, median PFS 23.7 months, median DOR 22.7 months and median OS 20.4 months. Anti-MM activity was not affected by any specific prior therapy consistent with the lack of cross-reactivity of selinexor with other anti-MM agents.

The high efficacy demonstrated here by XKd is on par with the efficacy demonstrated by currently recommended triplet combinations utilising carfilzomib and dexamethasone to treat RRMM. In RRMM patients treated with a median of 2 prior therapies (range 1–4), none of whom had previous treatment with daratumumab or had triple-class refractory MM, daratumumab, carfilzomib and dexamethasone (DKd) induced an ORR of 84% [31]. At a median follow-up of 16.6 months, DKd-induced median PFS was not reached [31]. In patients with RRMM with a median of six prior therapies (range 2–12), carfilzomib, pomalidomide and dexamethasone (KPd) induced an ORR of 50% [32]. At a median follow-up of 26.3 months, KPd induced a median PFS of 7.2 months and a median OS of 20.6 months [32]. While the current sample size is relatively small, these findings suggest that XKd is at least as active, and potentially more active in patients with anti-CD38 mAb refractory disease than other triplet combinations in heavily pretreated MM. These data position XKd as an optimal combination to address the unmet clinical need in the growing population of MM patients with prior therapy with an anti-CD38 mAb for which there is a scarcity of data and limited clinical efficacy with established therapy regimens. We do also note that most physicians treating progressing MM prefer to utilise agents in novel therapeutic classes, rather than switching to a second-generation compound in the same class, and hence XKd may provide a preferred regimen.

Two dose levels tested, 100 mg selinexor with 56 mg/m^2^ carfilzomib and 80 mg selinexor with 70 mg/m^2^ carfilzomib, both with 40 mg dexamethasone QW, were found to be intolerable in our patient cohort. However, the next lower level, 80 mg selinexor with 56 mg/m^2^ carfilzomib and 40 mg dexamethasone all QW, was identified as MTD and determined to be the RP2D. Additional dose levels including 60 mg selinexor (on days 1, 8, 15 and 22) with 70 mg/m^2^ carfilzomib and 80/100 mg selinexor with carfilzomib 70/56 mg/m2, both only on days 1, 8 and 15 of the 28-day cycles, are being evaluated in eight patients.

Mechanistically, the notable potency of the XKd regimen is well supported by preclinical studies [8, 14–17]: while PIs prevent the proteasome-mediated destruction of TSPs and other key regulatory/anti-cancer proteins in both the cytoplasm and the nucleus, they cannot control the geographic distribution of these molecules within the cell [33, 34]. Since TSPs and many other regulatory proteins only function in the nuclear compartment, resistance to PIs often occurs in the setting of elevated levels of XPO1—mediating the rapid nuclear export and functional inactivation of these key anti-cancer proteins. When XPO1 inhibitors are combined with PIs, levels of TSPs and other proteins are greatly increased, and they are restricted to the nuclear compartment leading to greatly enhanced anti-cancer activity [14, 17, 23].

The XKd safety profile was similar to that observed previously for each of the components [11, 18], with the most common non-haematologic TRAEs being nausea and fatigue, mostly Grades 1 and 2, reversible and manageable with dose modifications and/or supportive care. Prophylaxis with antivirals and intravenous (IV) hydration for carfilzomib, along with anti-nausea agents for selinexor, are important to optimise therapy. The most common haematological TRAEs were thrombocytopenia (with no associated bleeding) and anaemia, which were also reversible and manageable with dose modifications and/or supportive care. Discontinuation due to TRAEs occurred in 15.6% of all patients in the current study (median prior therapies = 4), and in 11.1% of patients at the RP2D, a rate comparable to that reported for other triplet combinations containing carfilzomib and dexamethasone with half the number of prior therapies (i.e. KRd = 16.8% [35], DKd = 22% [36]). Moreover, some of the most common AEs associated with carfilzomib, but not selinexor, were lower or similar in the current trial compared to other pivotal clinical trials testing carfilzomib in triplet combinations [35, 36]. Importantly, the XKd triplet is one of the simplest regimens to administer, requiring only the “backbone” weekly carfilzomib infusions (30 min of hydration, followed by 30 min of carfilzomib) with appropriate monitoring; the oral selinexor (and dexamethasone) can be given during the infusions. This is considerably simpler than double parenteral infusions (e.g. DKd) or parenteral-daily oral regimens (e.g. KPd).

In conclusion, the QW combination of oral selinexor 80 mg, dexamethasone 40 mg and IV carfilzomib 56 mg/m^2^ provided deep and durable responses in patients with heavily pretreated RRMM, of whom 37.5% had triple-class refractory MM and 53.1% had high-risk cytogenetics. Based on the preliminary results presented in this study in a more heavily pretreated population than those previously reported with carfilzomib-based triplets, QW XKd has compelling, durable activity, including in high-risk and triple-class refractory MM, and additional studies in both previously treated, as well as newly diagnosed MM, are warranted.

### Safety

Two of the first three patients enrolled into the selinexor 100 mg with 56 mg/m^2^ carfilzomib cohort experienced a DLT (Grade 3 thrombocytopenia and Grade 3 vomiting) (Table 1). Likewise, two of the first three patients enrolled into the selinexor 80 mg with 70 mg/m^2^ carfilzomib cohort experienced a DLT (Grade 4 thrombocytopenia ± pneumonia) (Table 1). No DLTs were observed in the first three patients enrolled into the 80 mg selinexor with 56 mg/m^2^ carfilzomib and 40 mg dexamethasone; three additional patients were enrolled in this cohort to confirm the tolerability and no DLTs occurred; therefore, this dose level and schedule were determined to be the MTD and the RP2D for the expansion phase. Assessment of DLTs in the dose-evaluation cohorts that were initiated after the RP2D is still ongoing.

All 32 patients received at least one dose of selinexor and were therefore included in safety analyses (Table 4). Among all patients, the most common (>50%) non-haematologic TRAEs (all; Grade 3 [no Grade 4 non-haematologic TRAEs were reported]) were nausea (71.9%; 6.3%) and fatigue (53.1%; 9.4%), were mostly grade 1/2 and manageable by dose modification and/or supportive care. Grade 3/4 non-haematological TRAEs that occurred in at least two patients were fatigue (9.4%, Grade 3), pneumonia (6.3%, Grade 3), nausea (6.3%, Grade 3) and hyperglycaemia (6.3%, one Grade 3 and one Grade 4). The most common haematologic TRAEs (all, Grade 3/4) were thrombocytopenia (71.9%, 46.9%) and anaemia (53.1%, 18.8% all Grade 3). Grade 4 thrombocytopenia occurred in seven patients (21.9%) and was not accompanied by bleeding in any patient. No cases of febrile neutropenia were reported. Overall, 17 (53.1%) patients had TRAEs leading to dose interruptions and 21 (65.6%) had TRAEs leading to dose reductions.

**Table 4:** Treatment-related adverse events occurring in ≥10% patients.

TRAEs	RP2D, *N* = 18; *n* (%)			All patients, *N* = 32; *n* (%)		
	Grade 3	Grade 4	Any Grade	Grade 3	Grade 4	Any grade
Haematopoietic						
Thrombocytopenia	6 (33.3)	3 (16.7)	14 (77.8)	8 (25.0)	7 (21.9)	23 (71.9)
Anaemia	2 (11.1)	0	11 (61.1)	6 (18.8)	0	17 (53.1)
Leukopenia	2 (11.1)	0	5 (27.8)	3 (9.4)	0	11 (34.4)
Neutropenia	1 (5.6)	0	6 (33.3)	2 (6.3)	0	9 (28.1)
Gastrointestinal						
Nausea	2 (11.1)	0	14 (77.8)	2 (6.3)	0	23 (71.9)
Decreased appetite	1 (5.6)	0	9 (50.0)	1 (3.1)	0	15 (46.9)
Dysgeusia	0	0	7 (38.9)	0	0	10 (31.3)
Diarrhoea	0	0	3 (16.7)	0	0	8 (25.0)
Constipation	0	0	2 (11.1)	0	0	6 (18.8)
Vomiting	0	0	4 (22.2)	1 (3.1)	0	5 (15.6)
Constitutional						
Fatigue	1 (5.6)	0	10 (55.6)	3 (9.4)	0	17 (53.1)
Weight decreased	0	0	8 (44.4)	0	0	13 (40.6)
Insomnia	0	0	2 (11.1)	0	0	4 (12.5)
Neurology						
Peripheral neuropathy	1 (5.6)	0	5 (27.8)	1 (3.1)	0	6 (18.8)
Other						
Dyspnoea	0	0	1 (5.6)	0	0	6 (18.8)
Hyperglycaemia	1 (5.6)	0	3 (16.7)	1 (3.1)	1 (3.1)	6 (18.8)
Blurred vision	0	0	3 (16.7)	0	0	6 (18.8)
Hyponatraemia	1 (5.6)	0	4 (22.2)	1 (3.1)	0	6 (18.8)
Hypomagnaesemia	0	0	3 (16.7)	0	0	5 (15.6)
Hypocalcaemia	0	0	2 (11.1)	0	0	4 (12.5)
Insomnia	0	0	2 (11.1)	0	0	4 (12.5)

Among patients at the RP2D (*N* = 18), the most common (≥50%) non-haematologic TRAEs (all; Grade 3) were nausea (77.8%; 11.1%), fatigue (55.6%; 5.6%) and decreased appetite (50.0%; 5.6%), were mostly Grade 1/2 and manageable by dose modification and/or supportive care. Nausea was the only Grade ≥3 non-haematological TRAEs that occurred in at least two patients (11.1%, G3). Most common haematologic TRAEs (all, Grade 3/4) were thrombocytopenia (77.8%, 50.0%) and anaemia (61.1%, 11.1% all G3). Grade 4 thrombocytopenia occurred in three patients (16.7%) and again was not accompanied by bleeding in any patient.

No patient had nausea or vomiting leading to discontinuation of any study drug. All patients received at least one prophylactic anti-nausea agent. Twenty-four (75%) received a 5-HT3 antagonist, and of those, 19 (79.2%) received at least one additional antiemetic medication. Ten patients (31.3%) received two or more additional antiemetic medications. Four (12.5%) patients received an additional appetite stimulant (i.e. megesterol, mirtazapine) and 11 (34.4%) patients received potassium chloride tablets. Six (18.8%) patients received transfusions (either red blood cell or platelet transfusions). Six (18.8%) patients received the thrombopoietin receptor agonists eltrombopag or romiplostim, one (3.1%) patient received filgrastim and two patients (6.3%) received an erythropoietin-stimulating agent.

Six patients had at least one serious AE) attributed to any of the study drugs: 2 pneumonia (6.3% of patients, Grade 3), 1 anaemia (Grade 3), 1 coronavirus disease 2019 (Grade 3), 1 cardiac failure (Grade 1), 1 fatigue (Grade 3), 1 influenza (Grade 3), 1 left ventricular failure (Grade 3), 1 encephalopathy (Grade 3), 1 *Pneumocystis jirovecii* pneumonia (Grade 3), 1 viral pneumonia (Grade 3), 1 pulmonary embolism (Grade 3), and 1 thrombocytopenia (Grade 4, no concurrent bleeding).

## Supplementary information

XKd Manuscript Supplemental

## Data Availability

Karyopharm Therapeutics agrees to share the individual participant data that underlie the results reported in this Article (after de-identification), including the study protocol. To gain access, data requestors should submit a request to medicalinformation@karyopharm.com.
