# Supplementary material for: Once weekly selinexor, carfilzomib and dexamethasone in carfilzomib non-refractory multiple myeloma patients
Source: Br J Cancer. 2021 Nov 20;126(5):718–25. doi: 10.1038/s41416-021-01608-2 (PMC8605887; doi:10.1038/s41416-021-01608-2)
Supplement: Supplementary file 1 — XKd Manuscript Supplemental [file 41416_2021_1608_MOESM1_ESM.docx]

**Supplemental Materials**

**Adverse event [AE] management guidelines/Supportive care (Clinical Study Protocol**

**KCP-330-017 Ver 11.0) – Concomitant Therapy**

**1. Required 5-HT3 Antagonists**

In order to minimize nausea, unless contraindicated, all patients must receive 5-HT3 antagonists (e.g., ondansetron 8 mg or equivalent), starting before the first dose of selinexor and continued 2-3 times daily thereafter, as needed. Alternative treatment may be provided if the patient does not tolerate 5-HT3 antagonists.

**2. Required Therapy for Non-Selinexor Drugs**

Patients receiving carfilzomib in Arm 6 should receive IV or PO dexamethasone approximately 30 minutes to 4 hours before each carfilzomib dose in Cycle 1, then as needed to help prevent infusion reactions, per carfilzomib’s labeling.

**3. Supportive Care**

Supportive measures for optimal medical care should be provided to patients during participation in this study. In ongoing clinical studies, the most common AEs reported as at least possibly related to selinexor have been low-grade nausea, fatigue, anorexia, thrombocytopenia, and vomiting. Most of these AEs can be managed effectively with dose modification and/or supportive care initiated prior to first dose.

In order to minimize nausea, unless contraindicated all patients should receive serotonin receptor subtype (5-HT3) antagonists (ondansetron 8 mg or equivalent), starting Q8 hours before each dosing and continue 2 -3 times daily for at least 2 days after dosing. Alternative antiemetic agents may be used if the patient does not tolerate or has inadequate antiemetic effect with 5-HT3 antagonists.

In addition, patients should receive olanzapine 2.5 mg PO daily starting on Day 1 and continuing for at least the first 2 months of the study. The olanzapine dose may be dose reduced due to side effects or stopped after 2 months of combination therapy period if nausea is well controlled. Patients may receive hydration prophylaxis, antibiotics, acid suppression (proton-pump inhibitors [PPI] and/or H2-blockers) and other treatments may be administered as follows:

- Appetite stimulants: megesterol acetate at a dose of 80-400 mg daily.
- Centrally acting agents: per National Comprehensive Cancer Network® [NCCN] Clinical Practice Guidelines®.
- Neurokinin-1 receptor (NK1R) antagonist: Aprepitant or equivalent should be considered and will be covered for selected patients who have severe nausea and vomiting.

**3.1. Infection**

Appropriate broad-spectrum IV antibiotics and antifungal agents should be started immediately in patients who develop fever or other signs of systemic infection. Selinexor should be suspended in any patient with Grade 4 infection or clinical sepsis (in the absence of documented infection) until the condition is stabilized. Selinexor can then be re-started at the same dose.

**3.2. Glucocorticoid Side Effects**

The management of common glucocorticoid side effects is well documented. Aggressive use of PPIs, anti-hypertensives, glucose-lowering drugs and other agents is strongly encouraged in order to maintain the use of dexamethasone in combination with selinexor in this study.

Patients with documented osteopenia or osteoporosis should continue to take dexamethasone with selinexor as indicated in the study. Standard precautions such as use of bisphosphonates should be instituted unless contraindicated.
